# Fenótipo Inflamatório por Imagem de OCT Coronária: Características Específicas Entre Lesões *De Novo* , Hiperplasia Intimal e Neoaterosclerose Intra-Stent

**DOI:** 10.36660/abc.20220045

**Published:** 2022-09-30

**Authors:** Luiz Fernando M Pinheiro, Stefano Garzon, José Mariani, Guy F. Almeida Prado, Adriano Mendes Caixeta, Breno Oliveira Almeida, Pedro Alves Lemos

**Affiliations:** 1 Hospital Israelita Albert Einstein São Paulo SP Brasil Hospital Israelita Albert Einstein , São Paulo , SP – Brasil; 2 Hospital das Clínicas Faculdade de Medicina Universidade de São Paulo São Paulo SP Brasil Instituto do Coração do Hospital das Clínicas da Faculdade de Medicina da Universidade de São Paulo , São Paulo , SP – Brasil; 3 Universidade Federal de São Paulo Escola Paulista de Medicina São Paulo SP Brasil Universidade Federal de São Paulo – Escola Paulista de Medicina , São Paulo , SP – Brasil

**Keywords:** Reestenose Coronária, Aterosclerose, Stents

## Abstract

**Fundamento:**

A estenose coronária pode ser causada por *de novo* aterosclerose, reestenose intra-stent e neoaterosclerose intra-stent, três entidades que se desenvolvem a partir de diversos meios fisiopatológicos.

**Objetivos:**

Este estudo tem como objetivo investigar, por meio da tomografia de coerência óptica (OCT), se as lesões coronarianas relacionadas a esses processos diferem em seu perfil inflamatório local.

**Métodos:**

Análise retrospectiva de pacientes com lesões coronárias diagnosticadas ou suspeitas que realizaram exames de OCT por motivos clínicos. Macrófagos e neovascularização intraplaca foram avaliados por OCT e utilizados como marcadores de inflamação local. O nível de significância < 0,05 foi adotado como estatisticamente significante.

**Resultados:**

Das 121 lesões, 74 eram *de novo* , 29 eram reestenose e 18 eram neoaterosclerose. Neovascularização foi encontrada em 65,8% das *de novo* , 10,3% na reestenose e 94,4% na neoaterosclerose (p<0,01 para todos). O volume de neovascularização foi diferente entre os tipos de lesão (950 vs. 0 vs. 6.220, respectivamente [valores medianos em 1000 x µm ^3^ /mm]; p<0,01 para todos), sendo significativamente maior na neoaterosclerose e menor na reestenose. A presença de macrófagos diferiu entre as lesões (95,9% em *de novo* vs. 6,9% em reestenose vs. 100% em neoaterosclerose [p<0,01 para todos]). Além disso, a intensidade da infiltração macrofágica foi diferente entre os tipos de lesão (2,5 vs. 0,0 vs. 4,5, respectivamente [valores medianos do escore de macrófagos]; p<0,01 para todos), significativamente maior na neoaterosclerose e menor na reestenose.

**Conclusões:**

Quando comparados pela OCT coronariana, *de novo* , reestenose intra-stent e neoaterosclerose apresentaram fenótipos inflamatórios marcadamente diferentes.

## Introdução

A doença aterosclerótica coronariana é uma causa prevalente de morbimortalidade em todo o mundo, frequentemente tratada com implante de stent. No entanto, é bem conhecido que um novo estreitamento do lúmen do stent pode ocorrer nos primeiros meses após a intervenção percutânea, um fenômeno conhecido como reestenose. ^1^ Ambas entidades ( *de novo* aterosclerose e reestenose intra-stent) originam-se marcadamente de mecanismos patogenéticos distintos. A formação de placa aterosclerótica é uma condição complexa, multifatorial e de longa duração modulada por múltiplos fatores de risco sistêmicos e locais. ^2^ Por outro lado, a reestenose intra-stent é secundária ao crescimento do tecido neointimal, uma resposta de cicatrização vascular desencadeada pela lesão do vaso após o implante do dispositivo. ^3 , 4^ Mais recentemente, a neoaterosclerose foi descrita como outra causa distinta de estreitamento do lúmen intra-stent. É largamente aceito que seja uma forma acelerada de formação de placa aterosclerótica, provavelmente induzida por uma resposta tecidual local sustentada pelo próprio suporte metálico do stent. ^5^ O acúmulo de células inflamatórias tem sido descrito como um evento central para o desenvolvimento da *de novo* aterosclerose ^2 , 6 , 7^ e reestenose intra-stent, ^8^ bem como para neo-aterosclerose. ^9^ Acredita-se que a inflamação local seja parte integrante dessas condições, funcionando como a etapa decisiva pela qual a parede do vaso é modificada dinamicamente à medida que o processo patológico progride. Até o momento, no entanto, tem sido mal descrito se os perfis inflamatórios variam de acordo com o tipo de condição subjacente e se as diferenças potenciais podem ser avaliadas por ferramentas clínicas. A tomografia de coerência óptica intravascular (OCT) fornece imagens in vivo próximas ao nível histológico, ^10^ que tem sido amplamente utilizada para investigar pacientes com doença arterial coronariana. ^11 - 13^ Além de medir quantitativamente parâmetros dimensionais, a OCT foi validada como uma ferramenta para avaliar as características qualitativas da parede do vaso, como tipo de componentes do tecido, acidentes de placa e formação de trombo. ^11 , 12^ Também de forma importante, a OCT demonstrou detectar com precisão a infitração de macrófagos ^10 , 14^ e a formação de neovasos intra-arteriais ^15^ dois achados associados à inflamação local subjacente. O presente estudo tem como objetivo investigar se aterosclerose, reestenose intra-stent e neo-aterosclerose diferem em seu fenótipo inflamatório (ou seja, presença e quantidade de macrófagos e neovasos) conforme avaliado por imagem de OCT.

## Métodos

### Seleção de pacientes

Realizamos uma busca no banco de dados de nossa instituição por pacientes que realizaram OCT coronariano em artérias coronárias nativas por condição clínica estável ou síndrome coronariana aguda, entre janeiro de 2012 e dezembro de 2019. Todas as execuções de OCT de cada paciente foram revisadas e selecionadas para análise final se apresentassem: i ) uma ou mais lesões ateroscleróticas *de novo* (definidas como um arco de placa ≥ 180°), ou ii) uma ou mais lesões em um stent previamente implantado (definido como pelo menos 300 µm de espessura de tecido no stent). Lesões no mesmo vaso foram consideradas discretas e contadas como tal, se separadas por um segmento normal maior que 10 mm. As lesões nas bordas do stent (5 mm proximal ou distal) não foram incluídas para análise. Além disso, o presente relato incluiu apenas lesões cujo exame de OCT foi realizado antes de qualquer intervenção. Este estudo foi aprovado pelo comitê de ética local e está de acordo com a Declaração de Helsinque.

### Aquisição de imagem e análise

A aquisição de imagem foi realizada usando técnicas padrão, durante a injeção de meio de contraste, conforme descrito em toda parte, ^16^ usando um sistema OCT no domínio da frequência (sistema C7 ou Ilumien OPTIS, catéteres de imagem C7 DragonFly ou DragonFly II, St. Jude Medical, St. Paul, MN ).

**Figura 1:**
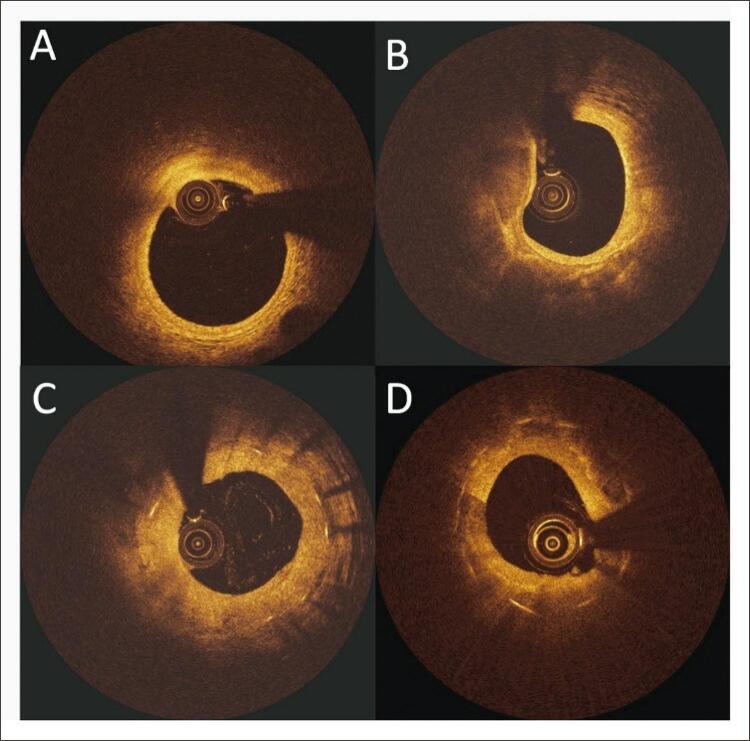
Imagem de OCT de uma artéria coronária normal (A), aterosclerose de novo (B),reestenose intra-stent (C) e neoaterosclerose (D). Reestenose intra-stent é caracterizada por uma aparência homogênea do tecido reestenótico neointimal enquanto a neoaterosclerose apresenta depósitos lipídicos e calcificados intra-stent.

Dois revisores independentes, cegos para qualquer informação clínica, realizaram as avaliações de todas as imagens de OCT. Qualquer discordância entre os revisores foi resolvida por consenso. As lesões foram classificadas como *de novo* , reestenose intra-stent ou neoaterosclerose intra-stent. Esta última lesão intra-stent foi diferenciada da anterior pela presença de depósitos calcificados ou lipídicos nas lesões neoateroscleróticas, em oposição à aparência homogênea do tecido reestenótico neointimal ( Figura 1 ). ^17 , 18^

**Figura 2:**
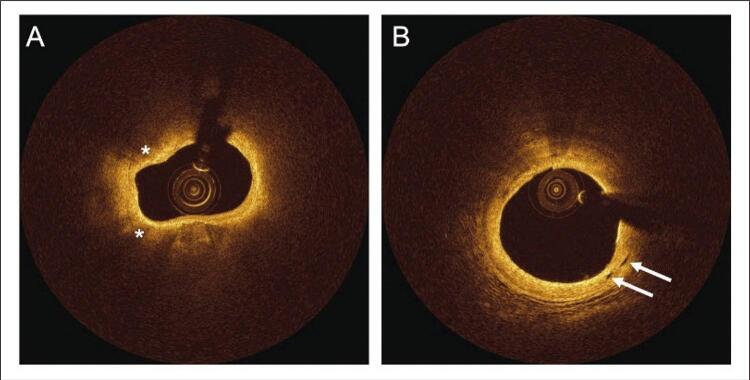
Imagens de OCT de infiltração de macrófagos e neovascularização. Os asteriscos brancos em A indicam imagens puntiformes ricas em sinal compatíveis com infiltração de macrófagos em imagens de OCT. As setas brancas em B indicam imagens intraplacas sem sinal compatíveis com neovascularização.

**Figura 3:**
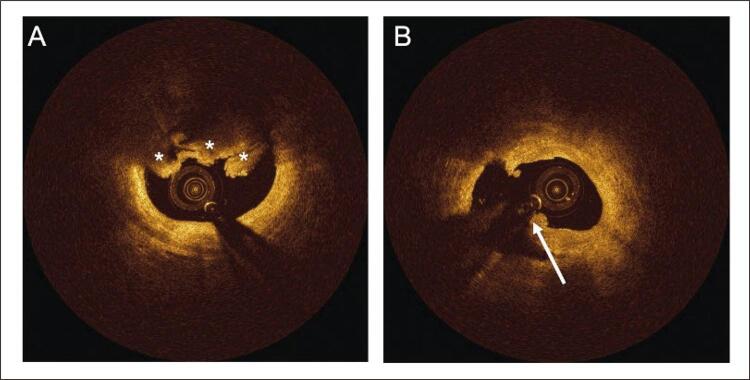
Imagens de OCT de trombo e placa rota. Os asteriscos brancos indicam trombo (A) e a seta branca indica ruptura da placa (B).

As lesões foram analisadas usando definições padrão, como sugerido em outros lugares. ^18 - 21^ O tecido lipídico foi definido como regiões de sinal pobre com bordas difusas mal definidas. O tecido fibroso foi definido como uma região com alto retroespalhamento e um sinal relativamente homogêneo. Depósitos calcificados foram identificados como estruturas sem sinal ou heterogêneas com bordas bem delineadas. O arco de cálcio foi medido no quadro com maior extensão de depósito calcário. Os macrófagos foram identificados pela presença de imagens pontilhadas ricas em sinal, distintas ou confluentes excedendo a intensidade do salpicado de fundo ( Figura 2A ); o acúmulo de macrófagos foi classificado usando uma pontuação de 0 a 4 em cada quadro e, em seguida, somado as graduações para toda a lesão. ^20^ A neovascularização foi definida como estruturas intraplacas sem sinal, sem conexão com o lúmen do vaso, medindo entre 50-300 µm e reconhecido em ≥ 3 quadros consecutivos ( Figura 2B ). ^18 , 21^ O volume de neovascularização foi calculado pela soma da área de neovascularização em cada quadro e, em seguida, aplicando a regra de Simpson. Tanto o acúmulo de macrófagos quanto o volume de neovascularização foram indexados pelo comprimento da placa, para permitir a comparação entre as lesões. Trombo foi definido como uma massa que se projeta no lúmen do vaso, tipicamente com contornos irregulares, descontínuo da superfície da parede do vaso ( Figura 3A ). Os fibroateromas de capa fina (FCF) foram definidos como uma região com arco lipídico máximo superior a 90° e espessura da capa <65 μm. A placa rôta foi definida pela presença de laceração da íntima, ruptura ou dissecção da capa ( Figura 3B ).

As análises OCT quantitativas off-line utilizaram um pacote de software dedicado (QIvus 3.0, Medis Medical, Holanda). Os parâmetros quantitativos incluíram o comprimento da placa, a área de secção transversal luminal mínima (AST) e a estenose luminal máxima (AST mínima ÷ média [distal e proximal] lúmen de referência AST). Para lesões intra-stent, a área neointimal (Stent AST menos lúmen AST) e a espessura neointimal (medida perpendicularmente da haste do stent ao lúmen) também foram calculadas.

### Análise estatística

As análises estatísticas foram realizadas usando SPSS 26.0 (IBM Corp. Armonk, NY, EUA). As variáveis categóricas são apresentadas como contagens e frequências e foram analisadas pelo teste de Qui-quadrado ou teste exato de Fisher quando apropriado. Para testar a normalidade de distribição, nós realizamos o teste Shapiro-Wilks. Variáveis contínuas não apresentaram distribuição normal, entretanto, seus resultados são apresentados como mediana e intervalo interquartil (IQR). Nós usamos o teste não paramétrico Kruskall-Wallis para comparações múltiplas. Quando necessário, comparações emparelhadas foram realizadas utilizando a abordagem por Dunn-Bonferroni. O nível de significância < 0.05 foi adotado como estatisticamente significante.

## Resultados

110 pacientes com 123 lesões tiveram pelo menos uma OCT de boa qualidade que mostrou uma lesão por inteiro antes de qualquer manipulação intervencionista e compuseram a população do presente estudo. A maioria dos pacientes eram do sexo masculino, acima de 60 anos, com múltiplos fatores de risco para doença arterial coronariana e apresentando síndrome coronariana aguda (SCA) na admissão hospitalar ( Tabela 1 ).

**Tabela 1:** Características clínicas e demográficas (n=110 pacientes)

Sexo masculino	88 (80,0)
Idade, anos	63 (56 – 71)
Hipertensão	75 (68,2)
Diabetes	33 (30,0)
Dislipidemia	90 (81,8)
Tabagismo (atual ou prévio)	61 (55,5)
História familiar de DAC	60 (54,5)
Síndrome coronária aguda	69 (62,7)

*Números são calculados (porcentagem) ou mediana (intervalo interquartil). DAC: doença arterial coronariana.*

**Figura 4:**
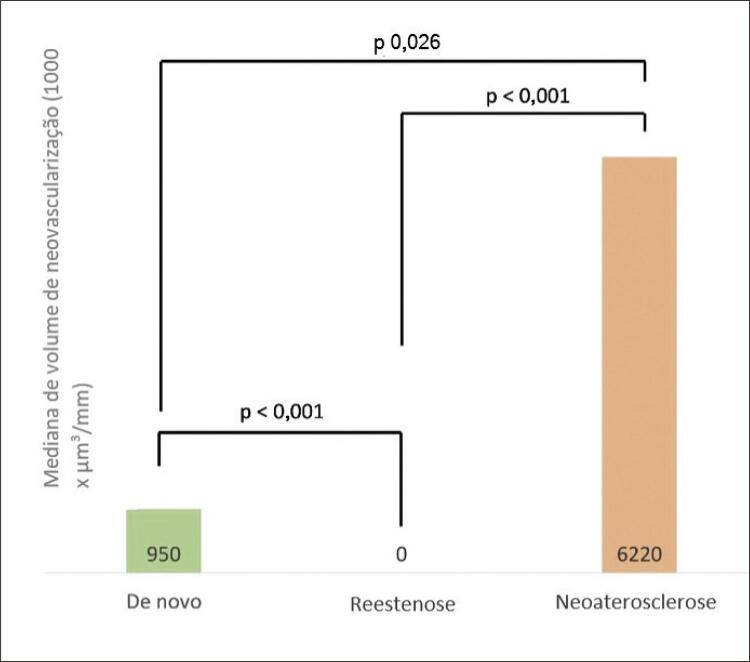
Volume de neovascularização por tipo de lesão.

Na Tabela 2 , nós apresentamos as características de acordo com o tipo de placa. A maioria das características foram diferentes entre os grupos. A neovascularização foi encontrada em 65,8% dos *de novo* , 10,3% na reestenose e 94,4% na neoaterosclerose (p<0,01 para todos) ( Tabela 2 ). Assim, o volume de neovascularização foi diferente entre os tipos de lesão (950 vs. 0 vs. 6.220, respectivamente [valores medianos em 1000 x µm ^3^ /mm]; p < 0,01 para todos), sendo significativamente maior na neoaterosclerose e menor na reestenose ( Figura 4 ).

**Tabela 2:** Características da OCT em lesões *de novo,* neoíntima e neoaterosclerose (n=121)

	*De novo* (n=74)	Reestenose intra-stent (n=29)	Neoaterosclerose Intra-stent (n=18)	p*
Calcificação	56 (75,7)	-	10 (55,6)	< 0,01
FCF	17 (23,3)	-	7 (38,9)	< 0,01
Ruptura de placa	10 (13,9)	0	7 (38,9)	< 0,01
Trombo	9 (12,5)	0	4 (22,2)	0,03
Neovascularização	48 (65,8)	3 (10,3)	17 (94,4)	< 0,01
Macrófago	71 (95,9)	2 (6,9)	18 (100)	< 0,01
Extensão de placa, em mm	24,1 (17,2-36,8)	25,8 (18,0-33,0)	23,5 (17,8-29,0)	0,9
Luminal mínimo AST, mm ^2^	2,42 (1,64-3,51)	2,72 (1,77-4,52)	1,85 (1,35-3,18)	0,07
Luminal Max. de estenose, %	65,5 (54,8-74,6)	45,7 (33,1-63,0)	66,2 (53,9-76,2)	<0,01
Max. IS tecido espessura, mm	-	0,74 (0,59-0,98)	1,13 (0,95-1,34)	< 0,01
Max. IS tecido AST, mm ^2^	-	3,54 (2,87-4,69)	4,96 (4,22-6,21)	<0,01
Neovasc. vol., 1000 x µm ^3^ /mm	950 (0-3400)	0 (0-0)	6220 (1250-13430)	< 0,01
Escore de macrófago	2,5 (0,9-4,9)	0,0 (0,0-0,0)	4,5 (3,1-7,3)	< 0,01

Números são calculados (porcentagem) ou mediana (intervalo interquartil). AST: área de secção transversal; IS: intra-stent; ADA: artéria descendente anterior; ACX: artéria circunflexa; Max: máximo; Neovasc: neovascularização; ACD: artéria coronária direita; FCF: fibroateroma de capa fina; Vol: volume. *Valor de p para comparação geral entre os grupos.

**Figura 5:**
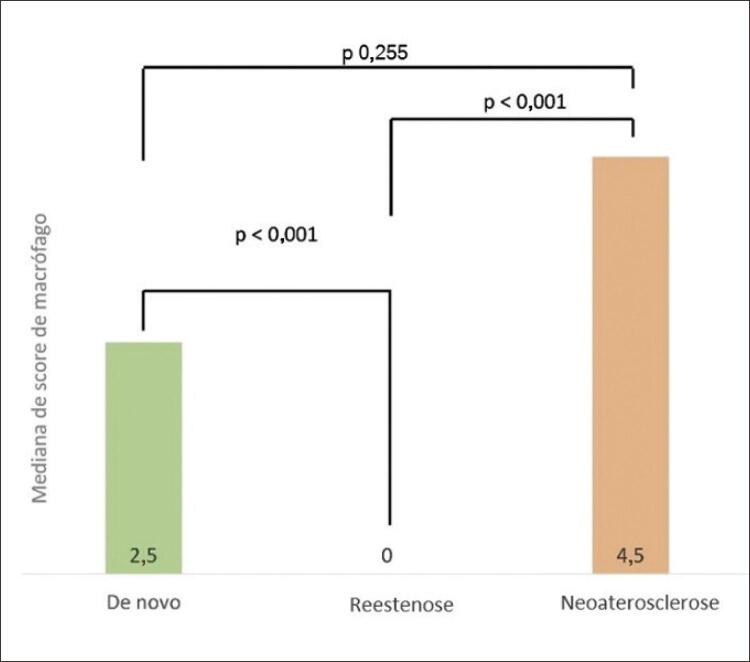
Escore de macrófagos por tipo de placa.

A presença de macrófagos diferiu entre as lesões (95,9% em *de novo* vs. 6,9% em reestenose vs. 100% em neoaterosclerose [p<0,01 para todos]). Além disso, a intensidade da infiltração macrofágica foi diferente entre os tipos de lesão (2,5 vs. 0,0 vs. 4,5, respectivamente [valores medianos do escore de macrófagos]; p<0,01 para todos) ( Figura 5 ), significativamente maior na neoaterosclerose e *de novo* aterosclerose e menor na reestenose ( Figura 5 ).

Quando comparados aos pacientes estáveis, pacientes com síndromes agudas tiveram maior presença de trombo (16,2 versus 2,4, p=0,029) e menor intensidade de infiltração de macrófagos (3,8 [1,2 – 5,9] versus 1,2 [0 – 3,6], p=0,008). Todos os outros achados de OCT (tipo de placa, presença de neovascularização, macrófagos, FCF, ruptura de placa e volume de neovascularização) não foram significativamente diferentes entre os grupos (p>0,05 para todos).

## Discussão

Nosso estudo comparou três diferentes causas de estenose coronária, a saber, lesões *de novo* , reestenóticas e neoateroscleróticas, e demonstrou diferenças marcantes entre elas em relação ao seu fenótipo inflamatório por OCT, avaliado pela presença e grau de acúmulo de macrófagos e neovasos intra-lesão.

A inflamação é o pilar para a compreensão desses três processos diferentes que causam estenose coronariana. A patogênese da aterosclerose coronariana nativa tem sido extensivamente investigada nas últimas décadas ^2^ e envolve múltiplas vias inflamatórias. No entanto, como o stent coronário é uma técnica um tanto recente, a reestenose intra-stent é uma entidade patológica que não existia anteriormente e ainda não é totalmente compreendida. Após a intervenção percutânea, ocorrem distúrbios do fluxo sanguíneo, migração e proliferação de células musculares lisas e fibroblastos para a íntima, causando deposição de matriz extracelular, colágeno, linfócitos e macrófagos. ^4 , 8 , 22^ Estímulo inflamatório contínuo causado pelas estruturas metálicas duradouras do stent também leva à reação de corpo estranho intra-placa, acelerando mudanças ateroscleróticas ^23^ e aumentando a presença de neovascularização. ^21^ Além disso, a maturação incompleta das células endoteliais devido a drogas anti-proliferativas liberadas pelos stents reduz a função de barreira normalmente executada pelo endotélio normal. ^24^ Tanto a presença aumentada de neovasos quanto o endotélio imaturo são provavelmente responsáveis por permitir um influxo acentuado de células inflamatórias no tecido neointimal. Ao contrário da aterosclerose do vaso nativo, que se desenvolve ao longo de décadas, ^2^ a neoaterosclerose é um processo aterosclerótico acelerado situado na parede do vaso anormalmente cicatrizado que pode ocorrer em poucos anos ou mesmo meses após o implante do stent, particularmente com stents farmacológicos. ^5^ Essas diferenças são observadas *in vivo* em nosso estudo, com a neoaterosclerose apresentando volumes de neovascularização e densidade de macrófagos significativamente maiores indicando uma alta atividade inflamatória nestas placas.

Acredita-se que a reestenose intra-stent devido à hiperplasia neointimal esteja limitada a um determinado período de tempo após o implante do stent ^25^ e foi geralmente considerada um evento um tanto benigno e estável, não frequentemente relacionado a eventos coronarianos agudos. ^26^ Mais recentemente, no entanto, tem sido observado que a reestenose intra-stent pode se apresentar como síndrome coronariana aguda em mais de 50% dos casos. ^27^ A neoaterosclerose provavelmente se desenvolve sobre a hiperplasia neointimal, ^21^ após modificações da placa infiltrada por lipídios e macrófagos, os quais são associados à ruptura da placa e eventos coronarianos agudos . Nossa população de estudo refletiu tais características, com placas neoateroscleróticas sendo significativamente mais propensas à ruptura do que placas *de novo* e reestenóticas, além de apresentarem maior espessura neointimal e tamanhos de lúmen, o que pode ser resultado de tais modificações da placa.

Nosso estudo tem várias limitações. Trata-se de um estudo exploratório, observacional, retrospectivo, com uma população altamente selecionada de indivíduos com alta carga de fatores de risco cardiovascular e doença arterial coronariana, e a maioria de nossa população (62,7%) foi composta por pacientes internados no hospital com síndromes coronarianas agudas. Assim, não é possível extrapolar esses achados para outros contextos clínicos. Além disso, pacientes com síndromes coronarianas agudas apresentaram baixos níveis de ilfiltração de macrófagos em nossa amostra. Este achado pode ser explicado pelo fato que pacientes agudos apresentaram significativamente mais trombos quando comparados aos pacientes estáveis tornando impossível em muitos casos o acesso à infiltração macrofágica nestas regiões. Embora com ausência de informações a respeito do tempo entre a inserção dos stents e a imagem de OCT, sobre o período ou tipo de stents implantados, em nossa opinião isso não prejudicou a interpretação de nossos achados, uma vez que estávamos analisando apenas as características da placa.

Não obstante, este é suposto ser um estudo gerador de hipóteses. A neoaterosclerose é uma importante causa de falência tardia do stent não reduzida com o uso de stents farmacológicos e tem impacto direto nos resultados das intervenções percutâneas coronarianas. ^28^ Fatores de risco como dislipidemia,tabagismo e filtração glomerular reduzida, todos fatores que regulam a inflamação sistêmica, estão associados com altos índices de neoaterosclerose. ^29 , 30^ Além disso, a inflamação por si só tem sido associada ao aumento do risco cardiovascular. ^2^ Novas evidências surgiram comprovando *in vivo* que a modulação da resposta inflamatória e controle dos fatores de risco podem reduzir as taxas de eventos cardiovasculares maiores ^7^ e reduzir o volume da placa aterosclerótica. ^31^ No entanto, esses efeitos ainda não foram comprovados nas reduções das taxas de hiperplasia neointimal e neoaterosclerose. Em publicação recente, ^32^ Hashikata et al. demonstraram que o uso de empaglifozina reduziu a hiperplasia neointimal em 12 meses em pacientes diabéticos quando comparado à terapia padrão de redução de glicose. As médias de espessura, volume e porcentagem neointimal foram significativamente menores no grupo empaglifozina. Curiosamente, essa redução foi independente de níveis reduzidos de glicose, sugerindo um possível mecanismo subjacente multifatorial. Atualmente, o estudo HUYGENS ^33^ incluiu pacientes com quadro de infarto do miocárdio sem supradesnivel do segmento ST que foram tratados com evolucumab ou placebo além de agressiva terapia com estatinas por 52 semanas e submetidos a procedimentos seriados de imagem com OCT e ultrasson intra-vascular. O grupo evolucumab atingiu um menor nível de LDL- colesterol e os achados de imagem incluiram um maior aumento da espessura da capa fibrosa, diminução do arco lipídico e redução da placa. Uma redução mais intensa do perfil lipídico com um precoce acréscimo do inibidor de PCSK9 às estatinas após um IAMSSST produz estabilização e regressão da aterosclerose coronária. A melhora dos resultados clínicos atingidos com níveis muito reduzidos de LDL-colesterol associado a mudanças no fenótipo da placa, prepara o caminho para estas novas opções de redução do perfil lipídico tornarem-se uma perspectiva na prevenção da neoaterosclerose intra-stent. Além disso, esforços estão sendo feitos na produção de stents com novas estruturas absorvíveis ^34^ e melhor liberação de drogas para modular a resposta tecidual, ^35^ permitindo assim uma regeneração endotelial mais fisiológica e reduzindo o substrato que origina a neoaterosclerose.

Até onde sabemos, este é o primeiro estudo comparando diretamente a inflamação da placa de aterosclerose de vasos nativos com hiperplasia neointimal e neoaterosclerose utilizando OCT. Em nosso entendimento, esses achados reforçam a importância da inflamação na patogênese da falência do stent, sugerindo que o futuro da ICP provavelmente está no fino ajuste da resposta tecidual não deixando para trás uma pegada metálica.

Estudos prospectivos adicionais com terapia lipídica agressiva, rígido controle da pressão arterial e glicemia, abandono do tabagismo e controle da inflamação podem modificar a evolução da neoaterosclerose.

## Conclusões

Em resumo, quando comparados usando OCT *, de novo* aterosclerose, reestenose intra-stent e neoaterosclerose intra-stent apresentaram fenótipos inflamatórios marcadamente diferentes (ou seja, volume de neovasos e quantificação de macrófagos).
