# Supplementary material for: Fenótipo Inflamatório por Imagem de OCT Coronária: Características Específicas Entre Lesões De Novo , Hiperplasia Intimal e Neoaterosclerose Intra-Stent
Source: Arq Bras Cardiol. 2022 Sep 30;119(6):931–7. [Article in Portuguese] doi: 10.36660/abc.20220045 (PMC9814796; doi:10.36660/abc.20220045)
Supplement: Translation

# Translation: Inflammatory Phenotype by OCT Coronary Imaging: Specific Features Among *De Novo* Lesions, In-Stent Neointima, and In-Stent Neo-Atherosclerosis

**Authors:** Luiz Fernando M Pinheiro, Stefano Garzon, José Mariani, Guy F. Almeida Prado, Adriano Mendes Caixeta, Breno Oliveira Almeida, Pedro Alves Lemos

**Affiliations:** 1 Brazil Hospital Israelita Albert Einstein, São Paulo, SP – Brazil; 2 Brazil Instituto do Coração do Hospital das Clínicas da Faculdade de Medicina da Universidade de São Paulo, São Paulo, SP – Brazil; 3 Brazil Universidade Federal de São Paulo – Escola Paulista de Medicina, São Paulo, SP – Brazil

**Keywords:** Coronary Restenosis, Atherosclerosis, Stents

## Abstract

**Background:**

Coronary stenosis can be caused de novo atherosclerosis, in-stent restenosis, and in-stent neoatherosclerosis, three entities that develop from a diverse pathophysiological milieu.

**Objective:**

This study aims to investigate, using optical coherence tomography (OCT), whether or not coronary lesions related to these processes differ in their local inflammatory profile.

**Methods:**

Retrospective analysis of patients with diagnosed or suspected coronary lesions who had undergone OCT imaging for clinical reasons. Macrophage and intra-plaque neovascularization were assessed by OCT and used as surrogates of local inflammation. A significance level of < 0.05 was adopted as statistically significant.

**Results:**

From the 121 lesions, 74 were de novo, 29 were restenosis, and 18 were neoatherosclerosis. Neovascularization was found in 65.8% of de novo, 10.3% in restenosis, and 94.4% in neoatherosclerosis (p<0.01 for all). The volume of neovascularization was different among lesion types (950 vs. 0 vs. 6220, respectively [median values in 1000 x µm^3^/mm]; p<0.01 for all), which were significantly higher in neoatherosclerosis and lower in restenosis. The presence of macrophages differed among the lesions (95.9% in de novo vs. 6.9% in restenosis vs. 100% in neoatherosclerosis [p<0.01 for all]). Moreover, the intensity of macrophagic infiltration was different among lesion types (2.5 vs. 0.0 vs. 4.5, respectively [median values of macrophage score]; p<0.01 for all), significantly higher in neoatheroscleosis and lower in restenosis.

**Conclusion:**

When compared using coronary OCT, de novo atherosclerosis, in-stent restenosis, and neoatherosclerosis presented markedly different inflammatory phenotypes.

## Introduction

Coronary atherosclerotic disease is a ubiquitous cause of morbimortality worldwide, frequently treated with stent implantation. However, it is well known that re-narrowing of the lumen of the stent may occur within the first months following percutaneous intervention, a phenomenon referred to as restenosis.^1^ Both entities (i.e. *de novo* atherosclerosis and in-stent restenosis) originate from markedly distinct pathogenetic mechanisms. Atherosclerotic plaque formation is a complex, multifactorial, long-lasting condition modulated by multiple systemic and local risk factors.^2^ On the other hand, in-stent restenosis is secondary to neointimal tissue growth, a vascular healing response triggered by vessel injury following device implantation.^3 , 4^ More recently, neoatherosclerosis has been described as another distinct cause of in-stent lumen narrowing. It is largely believed to be an accelerated form of atherosclerotic plaque formation probably induced by a sustained local tissular response to the stent metallic scaffold itself.^5^ The accumulation of inflammatory cells has been described as a central event for the development of *de novo* atherosclerosis^2 , 6 , 7^ and in-stent restenosis,^8^ as well as for neo-atherosclerosis.^9^ Local inflammation is believed to be an integral part of those conditions, functioning as the decisive step through which the vessel wall is dynamically modified as the pathologic process progresses. To date, however, it has been poorly described whether inflammatory profiles do vary according to the underlying type of condition, and if the potential differences can be assessed by clinical tools. Intravascular optical coherence tomography (OCT) provides *in vivo* near histology-level imaging,^10^ which has been largely used to investigate patients with coronary artery disease.^11 - 13^ In addition to quantitatively measuring dimensional parameters, OCT has been validated as a tool to assess qualitative characteristics of the vessel wall, such as tissue type components, plaque accidents, and thrombus formation.^11 , 12^ Also importantly, OCT has been shown to accurately detect macrophage infiltration^10 , 14^ and intra-wall neovessel formation,^15^ two features associated with subjacent local inflammation. The present study aims at investigating whether atherosclerosis, in-stent restenosis, and neo-atherosclerosis differ in their inflammatory phenotype (i.e., macrophage and neovessel presence and quantity) as assessed by OCT imaging.

## Methods

### Patient selection

We conducted a search in our institution’s database for patients who had undergone coronary OCT in native coronary arteries for clinical indications, between January of 2012 and December of 2019. All OCT runs from every patient were revised, and selected for final analysis if presenting: i) one or more *de novo* atherosclerotic lesions (defined as a plaque arc ≥ 180°), or ii) one or more lesions in a previously implanted stent (defined as at least 300 µm of in-stent tissue thickness). Lesions in the same vessel were considered discrete, and counted as such, if separated by a normal segment longer than 10 mm. Lesions at the stent edges (5-mm proximal or distal) were not included for analysis. Also, the present report only included lesions which OCT examination was performed prior to any intervention. This study was approved by the local ethics committee and is in accordance with the Declaration of Helsinki.

### Image acquisition and analysis

Image acquisition was performed using standard techniques, during injection of contrast media as described elsewhere,^16^ using a frequency-domain OCT system (C7 or Ilumien OPTIS system, C7 DragonFly or DragonFly II imaging catheters, St. Jude Medical, St. Paul, MN).

Two independent reviewers blinded to any clinical information performed the evaluations of all OCT images. Any disagreement between the reviewers was resolved by consensus. Lesions were classified as *de novo* , in-stent restenosis, or in-stent neoatherosclerosis. The latter in-stent lesion was differentiated from the former by the presence of calcific or lipidic deposits in neoatherosclerotic lesions, as opposed to the homogenous appearance of the neointimal restenotic tissue ( Figure 1 ).^17 , 18^

**Figure 1:** OCT images of a normal coronary artery (A), de novo atherosclerosis (B), in-stent restenosis (C) and neoatherosclerosis (D). In-stent restenosis is characterized by the homogenous appearance of the neointimal restenotic tissue, while neoatherosclerosis presents in-stent lipidic and calcific deposits.

Lesions were analyzed using standard definitions, as suggested elsewhere.^18 - 21^ Lipid tissue was defined as signal-poor regions with poorly defined, diffuse borders. Fibrous tissue was defined as a region with high backscattering and a relatively homogeneous signal. Calcific deposits were identified as signal-poor or heterogeneous structures with sharply delineated borders. The arc of calcium was measured at the frame with the largest extension of calcific deposit. Macrophages were identified by the presence of signal-rich, distinct, or confluent punctate images exceeding the intensity of background speckle noise ( Figure 2A ); macrophage accumulation was graded using a score from 0 to 4 in each frame and then summed the gradings for the whole lesion.^20^ Neovascularization was defined as no-signal, intra-plaque structures without connection to the vessel lumen measuring between 50-300 µm and recognized in ≥ 3 consecutive frames ( Figure 2B ).^18 , 21^ The volume of neovascularization was calculated by summing the area of neovascularization in each frame and then applying Simpson’s rule. Both macrophage accumulation and volume of neovascularization were indexed by plaque length, to allow for comparison among lesions. Thrombus was defined as a mass protruding into the vessel lumen, typically with irregular contours, discontinuous from the surface of the vessel wall ( Figure 3A ). Thin-cap fibroatheromas (TCFA) were defined as a region with maximal lipid arc more than 90°and cap thickness <65 μm. Ruptured plaque was defined by the presence of intimal tearing, disruption, or dissection of the cap ( Figure 3B ).

**Figure 2:** OCT images of macrophage infiltration and neovascularization. The white asterisks in A indicate signal-rich, punctate images compatible with macrophage infiltration in OCT images. The white arrows in B indicate no-signal, intra-plaque images compatible with neovascularization.

**Figure 3:** OCT images of thrombus and ruptured plaque. The white asterisks indicate thrombus (A) and the white arrow indicates plaque rupture (B).

Off-line quantitative OCT analyses utilized a dedicated software package (QIvus 3.0, Medis Medical, The Netherlands). Quantitative parameters included plaque length, minimal luminal cross-sectional area (CSA), and maximal luminal stenosis (minimal CSA ÷ mean [distal and proximal] reference lumen CSA). For *in-stent* lesions, neointimal area (stent CSA minus lumen CSA) and neointimal thickness (measured perpendicularly from stent strut to lumen) were also calculated.

### Statistical analysis

Statistical analyses were performed using SPSS 26.0 (IBM Corp. Armonk, NY, USA). Categorical variables are presented as counts and frequencies and were analyzed using Chi-square or Fisher’s exact test when appropriate. To test the normality of distribution, we performed a Shapiro-Wilks test. Continuous variables did not present normal distribution; therefore, their results are presented as median and interquartile range (IQR). We used the non-parametric Kruskall-Wallis test for multiple comparisons. When needed, pairwise comparisons were performed using the Dunn-Bonferroni approach. A significance level of < 0.05 was adopted as statistically significant.

## Results

Out of a total of 499 patients found in our database, 110 patients had at least one good-quality OCT run that imaged an entire lesion prior to any interventional manipulation, and comprised the present study population. Most patients were males, over 60 years of age, with multiple risk factors for coronary artery disease and presenting with acute coronary syndrome (ACS) upon hospital admission ( Table 1 ).

**Table 1:** Demographic and clinical characteristics (n=110 patients)

Males	88 (80.0)
Age, years	63 (56 – 71)
Hypertension	75 (68.2)
Diabetes	33 (30.0)
Hyperlipidemia	90 (81.8)
Smoking (current or former)	61 (55.5)
Family history of CAD	60 (54.5)
Acute coronary syndrome	69 (62.7)

*Numbers are counts (percentage) or median (interquartile range). CAD: coronary artery disease.*

In Table 2 , we present the characteristics among the different types of plaque. Most characteristics were different among the groups. Neovascularization was found in 65.8% of *de novo* , 10.3% in restenosis, and 94.4% in neoatherosclerosis (p<0.01 for all) ( Table 2 ). Accordingly, the volume of neovascularization was different among lesion types (950 vs. 0 vs. 6220, respectively [median values in 1000 x µm^3^ /mm]; p<0.01 for all), being significantly higher in neoatherosclerosis and lower in restenosis ( Figure 4 ).

**Table 2:** OCT characteristics of de novo, neointimal, and neoatherosclerotic lesions (n=121)

	De novo (n=74)	In-stent restenosis (n=29)	In-stent neoatherosclerosis (n=18)	p*
Calcification	56 (75.7)	-	10 (55.6)	< 0.01
TCFA	17 (23.3)	-	7 (38.9)	< 0.01
Plaque rupture	10 (13.9)	0	7 (38.9)	< 0.01
Thrombus	9 (12.5)	0	4 (22.2)	0.03
Neovascularization	48 (65.8)	3 (10.3)	17 (94.4)	< 0.01
Macrophage	71 (95.9)	2 (6.9)	18 (100)	< 0.01
Plaque length, in mm	24.1 (17.2-36.8)	25.8 (18.0-33.0)	23.5 (17.8-29.0)	0.9
Minimal luminal CSA, mm^2^	2.42 (1.64-3.51)	2.72 (1.77-4.52)	1.85 (1.35-3.18)	0.07
Max. luminal stenosis, %	65.5 (54.8-74.6)	45.7 (33.1-63.0)	66.2 (53.9-76.2)	<0.01
Max. IS tissue thickness, mm	-	0.74 (0.59-0.98)	1.13 (0.95-1.34)	< 0.01
Max. IS tissue CSA, mm^2^	-	3.54 (2.87-4.69)	4.96 (4.22-6.21)	<0.01
Neovasc. vol., 1000 x µm^3^/mm	950 (0-3400)	0 (0-0)	6220 (1250-13430)	< 0.01
Macrophage score	2.5 (0.9-4.9)	0.0 (0.0-0.0)	4.5 (3.1-7.3)	< 0.01

Numbers are counts (percentage) or median (interquartile range). CSA: cross sectional area; IS: in-stent; LAD: left anterior descending artery; LCx: left circumflex artery; Max: maximal; Neovasc: neovascularization; RCA: right coronary artery; TCFA: Thin-cap fibroatheroma; Vol: volume. *P-value for the overall comparison among the groups.

**Figure 4:** Neovascularization volume by lesion type.

The presence of macrophages differed among the lesions (95.9% in *de novo* vs. 6.9% in restenosis vs. 100% in neoatherosclerosis [p<0.01 for all]). Also, the intensity of macrophagic infiltration was different among lesion types (2.5 vs. 0.0 vs. 4.5, respectively [median values of macrophage score]; p<0.01 for all) ( Figure 5 ), significantly higher in neoatheroscleosis and lower in restenosis ( Figure 5 ).

**Figure 5:** Macrophage score by plaque type.

When compared to stable patients, acute patients had more thrombus (16.2% versus 2.4%, p = 0.029) and lower intensity of macrophage infiltration (3.8 [1.2 – 5.9] versus 1.2 [0 – 3.6], p = 0.008). All other OCT features (plaque type; presence of neovascularization, macrophage, TCFA and plaque rupture; and volume of neovascularization) were not significantly different between the groups (p > 0.05 for all).

## Discussion

Our study compared three different causes of coronary narrowing, namely de novo, restenotic and neoatherosclerotic lesions, and demonstrated marked differences among them in relation to their inflammatory phenotype by OCT, assessed by the presence and degree of macrophage accumulation and intra-lesion neovessels.

Inflammation is the cornerstone for understanding these three different processes that cause coronary stenosis. Pathogenesis of native coronary atherosclerosis has been extensively investigated in the last decades^2^ and involves multiple inflammatory pathways. However, since coronary stenting is a somewhat recent technique, in-stent restenosis is a pathological entity that did not exist previously and is not entirely understood yet. Following percutaneous intervention, blood flow disturbances, migration and proliferation of smooth muscle cells and fibroblasts into the intima ensues, causing deposition of extracellular matrix, collagen, lymphocytes and macrophages.^4 , 8 , 22^ Continuous inflammatory stimulus caused by the enduring metallic structures of the stent also leads to intra-plaque foreign-body reaction, accelerating atherosclerotic changes^23^ and increasing the presence of neovascularization.^21^ Also, incomplete maturation of endothelial cells due to the anti-proliferative drugs eluted by the stents impairs the barrier function usually carried out by the normal endothelium.^24^ Both increased neo-vessel presence and immature endothelium are likely responsible for allowing a steep inflow of inflammatory cells into the neointimal tissue. Unlike native vessel atherosclerosis, that develops over decades,^2^ neoatherosclerosis is an accelerated atherosclerotic process set in an abnormally healed vessel wall that can occur in a few years or even months following stent implantation, particularly with drug-eluting stents.^5^ These differences are observed *in vivo* in our study, with neoatherosclerosis presenting significantly larger neovascularization volumes and macrophage density when compared to both *de novo* and restenotic lesions.

In-stent restenosis due to neointimal hyperplasia is believed to be limited to a certain timeframe following stent implantation^25^ and was generally considered to be a somewhat benign, stable event, not frequently related to acute coronary events.^26^ More recently, however, it has been observed that in-stent restenosis may present as acute coronary syndrome in more than 50% of the cases.^27^ Neoatherosclerosis probably develops itself upon neointimal hyperplasia,^21^ following plaque modifications that infiltrate lipids and macrophages, which are associated with plaque rupture and acute coronary events. Our study population reflected such characteristics, with neoatherosclerotic plaques being significantly more prone to rupture than *de novo* and restenotic plaques, as well as presenting larger neointimal thickness and lumen sizes, which can be the result of such plaque modifications.

Our study has several limitations. It is an exploratory, observational, retrospective study, with a highly selected population of individuals with a high burden of cardiovascular risk factors and coronary artery disease, and most of our population (62.7%) was comprised of patients admitted to the hospital with acute coronary syndromes. Accordingly, it is not possible to extrapolate these findings to other clinical settings. Also, patients with acute coronary syndromes presented lower levels of macrophage infiltration in our sample. This finding may be explained by the fact that acute patients had significantly more thrombus when compared to stable patients, thus hindering the assessment of macrophage infiltration in these regions impossible in most cases. Although we had no information regarding the age or type of the stents implanted, in our opinion this was not detrimental to the interpretation of our findings, considering we were solely analyzing plaque characteristics.

Notwithstanding, this is supposed to be a hypothesis-generating study. Neoatherosclerosis is an important cause of late stent failure not reduced with the use of drug eluting stents and has a direct impact on the outcomes of coronary percutaneous interventions.^28^ Risk factors such as hyperlipidemia, smoking and impaired renal function, all of which up-regulate systemic inflammation, have been associated with higher rates of neoatherosclerosis.^29 , 30^ Additionally, inflammation by itself has been linked with increased cardiovascular risk.^2^ New evidence has emerged proving *in vivo* that modulation of the inflammatory response and risk factors control can reduce the rates of major cardiovascular events^7^ and reduce atherosclerotic plaque volume.^31^ Nonetheless, these effects are yet to be proven to reduce neointimal hyperplasia and neoatherosclerosis rates. In a recent article,^32^ Hashikata et al. demonstrated that the use of empaglifozin reduced neointimal hyperplasia at 12 months in diabetic patients when compared to standard glucose-lowering therapy. Mean neointimal thickness, volume and percentage were significantly lower in the empaglifozin group. Interestingly, this reduction was independent of lower glucose levels, suggesting a possible multi-factorial underlying mechanism. The current HUYGENS trial^33^ included patients with non-ST segment elevation myocardial infarction who were treated with evolocumab or placebo in addition to intensive statin therapy for 52 weeks and underwent serial OCT and intravascular ultrasound imaging. The evolocumab group achieved lower LDL-C levels and imaging features that included a greater increase in minimum fibrous cap thickness, decrease in maximum lipid arc and plaque regression. More intensive lipid lowering with early addition of a PCSK9 inhibitor to statins after a NSTEMI produces stabilization and regression of coronary atherosclerosis. The improved clinical outcomes achieved with very low LDL-C levels associated with changes in plaque phenotype pave the way for these new lipid-lowering options become a perspective to prevent intra-stent neoatherosclerosis. Also, efforts are being made to produce stents with novel absorbable scaffolds^34^ and better drug delivery to modulate tissular response,^35^ thus allowing a more physiologic endothelial regeneration and reducing the substrate that originates neoatherosclerosis.

To the best of our knowledge, this is the first study directly comparing plaque inflammation of native vessel atherosclerosis with neointimal hyperplasia and neoatherosclerosis using OCT. In our understanding, these findings stress the importance of inflammation in the pathogenesis of stent failure, suggesting that the future of PCI probably lies in fine-tuning tissular response and not leaving a metallic footprint behind.

Further prospective studies with aggressive lipid-lowering therapy, blood pressure and glucose control, smoking cessation, and control of inflammation may modify the evolution of neoatherosclerosis.

## Conclusions

In summary, when compared using OCT, *de novo* atherosclerosis, in-stent restenosis and in-stent neoatherosclerosis presented markedly different inflammatory phenotypes (i.e., neovessel volume and macrophage quantification).
